# Circulating miRNA profiles in COVID-19 patients and meta-analysis: implications for disease progression and prognosis

**DOI:** 10.1038/s41598-023-48227-w

**Published:** 2023-12-08

**Authors:** Liangliang Gao, Espoir M. Kyubwa, Mark A. Starbird, Jesus Diaz de Leon, Michelle Nguyen, Claude J. Rogers, Naresh Menon

**Affiliations:** ChromoLogic, LLC, Monrovia, CA USA

**Keywords:** Infectious diseases, Biomarkers

## Abstract

We compared circulating miRNA profiles of hospitalized COVID-positive patients (n = 104), 27 with acute respiratory distress syndrome (ARDS) and age- and sex-matched healthy controls (n = 18) to identify miRNA signatures associated with COVID and COVID-induced ARDS. Meta-analysis incorporating data from published studies and our data was performed to identify a set of differentially expressed miRNAs in (1) COVID-positive patients versus healthy controls as well as (2) severe (ARDS^+^) COVID vs moderate COVID. Gene ontology enrichment analysis of the genes these miRNAs interact with identified terms associated with immune response, such as interferon and interleukin signaling, as well as viral genome activities associated with COVID disease and severity. Additionally, we observed downregulation of a cluster of miRNAs located on chromosome 14 (14q32) among all COVID patients. To predict COVID disease and severity, we developed machine learning models that achieved AUC scores between 0.81–0.93 for predicting disease, and between 0.71–0.81 for predicting severity, even across diverse studies with different sample types (plasma versus serum), collection methods, and library preparations. Our findings provide network and top miRNA feature insights into COVID disease progression and contribute to the development of tools for disease prognosis and management.

## Introduction

The global COVID-19 pandemic, caused by the severe acute respiratory syndrome coronavirus 2 (SARS-CoV-2), has resulted in significant morbidity and mortality worldwide, with over 765 million confirmed cases and 6.9 million deaths reported^1^. Severe cases of COVID-19 can lead to acute respiratory distress syndrome (ARDS), which is associated with a higher incidence of death^2^. Despite the widespread availability of effective vaccines and treatments for COVID-19 across many countries, it remains imperative to accurately predict disease severity and identify enriched biological pathways. These efforts continue to be crucial in optimizing treatment strategies and enhancing patient outcomes.

MicroRNAs (miRNAs) are small (~ 22 nt) noncoding RNAs^3,4^ that play important roles in various biological and pathological processes and have gained momentum and been used as biomarkers for several cancers and other diseases^5–8^. Circulating miRNAs are promising biomarkers for disease prognosis applications, as they are transcriptome-regulating biomolecules that are stably packaged in vesicles or protein complexes and accessible via routine blood draw.

A PubMed search using the keywords "circulating microRNA" and "COVID" resulted in over 40 publications, with sample sizes ranging from 20 to over two hundred individuals. While all these publications contribute to the understanding of the role of miRNAs in COVID-19, only a selected few were chosen to compare with our study. We focus on studies with (i) more recent publication dates (mostly 2022) that offer the advantage of capturing the most up-to-date knowledge in the field, (ii) similar categories of patients to our research population, and (iii) with publicly available raw miRNA sequencing data to ensure transparency and reproducibility of the findings. While this process may not have been exhaustive; we believe that the chosen studies provide sufficient diversity, patient age, ethnicity, population, and trial conditions to identify miRNAs that are robustly dysregulated by COVID-19 infection.

For instance, Zeng et al.^9^ conducted a comprehensive analysis of miRNA profiles from 236 individuals with varying clinical presentations of SARS-CoV-2 infection. They proposed that hsa-miR-370, hsa-miR-1246, hsa-miR-483 and more are associated with COVID-19 disease infection, and that hsa-miR-625 and miR-143 and more are associated with disease severity (severe vs moderate COVID). Furthermore, the study revealed the importance of NF-κB signaling and interleukin pathways in the progression of COVID-19. Gutmann et al.^10^ analyzed miRNA-seq data from 47 subjects including healthy controls, non-severe, and severe COVID-19 patients. They identified hsa-miR-150, hsa-miR-21 to be involved in COVID-19 disease infection and hsa-miR-122 and hsa-miR-133 to be associated with COVID severity. Garcia et al.^11^ identified over 100 differentially expressed miRNAs and narrowed down to a key miRNA, hsa-miR-369, that could distinguish COVID-19 disease severity among 28 patients. Togami et al.^12^ performed mRNA and miRNA sequencing of 62 individuals and identified key miRNA features including hsa-miR-150 and has-miR-143 for COVID disease infection and highlighted the importance of interferon pathway in COVID-19 pathogenicity. While these individual studies offer valuable insights, we decided to perform a meta-analytical approach by aggregating and analyzing findings from different studies (including our own). Previously, meta-analysis have been utilized to improve diagnosis and prognosis of multiple injuries and diseases^13–15^. The goal of our work is to identify common COVID-19 molecular signatures by reconciling discrepancies or variations between individual studies.

We conducted a study to identify circulating miRNAs as potential biomarkers for predicting COVID-19 disease severity. The circulating miRNA profile of three groups were compared; 77 patients with confirmed COVID-19 but no ARDS (11 did not survive the disease), 27 patients with confirmed COVID-19 and ARDS (11 did not survive the disease), and 18 roughly age- and sex-matched healthy volunteers without COVID-19 (collected before the pandemic). We identified differentially expressed miRNAs and performed gene ontology enrichment analysis (GOEA) of the genes regulated by the differentially expressed miRNA to build an understanding of the underlying biological processes associated with COVID-19. The identified biomarkers were found to regulate genes associated with interleukin expression, TLR pathways, T cell proliferation, and intrinsic apoptosis as well as virus genome pathways.

Furthermore, through the utilization of a meta-analysis approach, we combined our findings with other studies to identify a shared set of dysregulated miRNAs. Notably, we observed a significant down-regulation of a large cluster of miRNAs located on chromosome 14 (14q32), comprising over 90 members. We built machine learning models based on meta-analysis results combined with an exhaustive feature selection tool^16^ that was able to predict COVID-19 disease and severity across multiple independently published studies. Our results suggest a robust method for building miRNA-based models for disease diagnosis and prognosis and highlight overlapping roles of different miRNA biomarkers. In this paper, we present our findings and discuss the potential implications for developing new therapies and companion diagnostics for COVID-19.

## Results

### Patient demographics and clinical chemistry

A summary of the ethnic, sex, and age distribution across three cohorts (normal, severe ARDS + COVID and moderate ARDS- covid patients in our study as well as comparisons to seven published studies are provided in Table 1 and Supplementary Tables S1. In our study, the median ages for the normal, moderate COVID, and severe COVID groups were 62, 70, and 66 years, respectively. The percentage of females in the normal, moderate COVID, and severe COVID groups were 42, 44, and 30%, respectively. Our study’s population age and sex distributions align with the range of values reported in other studies^9–11^.

**Table 1:** Subject demographics and main findings of COVID miRNA studies.

Studies	Gao current	Togami^12^	Zeng^9^	Fernández-Pato^17^	Gutmann^10^	Farr^18^	Garcia‑Giralt^11^	Madè^19^
Sample type	Plasma	Serum	Plasma	Plasma	Plasma	Plasma	Serum	Plasma
Country	US	Japan	China	Spain	UK	Australia	Spain	Italy
Ethnicity-main	Caucasian Black	Asian	Asian	Caucasian Hispanic	Caucasian	Caucasian	Caucasian	Caucasian
Year	–	2022	2022	2022	2022	2021	2022	2022
Sample size (HC; MOD; SEV)	18	21	61	13	12	10	NA	NA
	77	41	68	64	18	10	13	44
	27	NA	48	32	18	NA	15	NA
Age (HC; MOD; SEV)	62 (47–67)	–	42 (29–66)	–	40 (30–46)	–	NA-NA-NA	–
	70 (60–77)		53 (43–60)		55 (36–66)		50 (43–54)	
	66 (57–74)		64 (55–69)		58 (39–66)		51 (48–55)	
Gender-F(%)	8 (42%)	NA	6 (38%)	–	6 (58%)	–	NA-NA-NA	–
	34 (44%)		35(67%)		8 (44%)		6 (54%)	
	8 (30%)		32(48%)		10 (58%)		6 (40%)	
AST	17.5 (12–24)	–	–	–	–	–	–	–
	28 (21–43)							
	30 (22–55)							
ALT	13 (11–18.2)	–	NA	NA	29 (17,40)	–	–	–
	25.5 (15.8–42)				21 (17, 43)			
	33 (24.5–54)				52 (33, 65)			
Neutrophil	4.2 (2.9–5)	–	–	–	3.6 (2.8–4.5)	–	–	–
	4.9(3.5–7)				2.9 (2–5.4)			
	6 (4–12)				7.3 (4.9–9)			
Lymphocytes	1.7 (1.3–2.2)	–	–	–	1.8 (1.6–2.1)	–	–	–
	1 (0.6–1.5)				1.1 (0.7–1.3)			
	1 (0.8–1.7)				0.5 (0.4–0.7)			
Platelets	216 (193.5–305.2)	–	–	–	214.0 (188.0–244.0)	–	–	–
	229 (188.5–330.5)				208 (157–257)			
	293 (207.5–384)				274 (158–339)			
MiRNA data available	Reads, Counts, log2FC	Counts	Counts log2FC	log2FC	Counts log2FC	Reads, log2FC	Counts, log2FC	Counts
Model AUC	0.81–0.93* 0.71–0.81*^†^	0.5–0.75	0.9–0.99 0.72–0.99^**†**^	0.88–0.97 (survival)	0.87–0.94^**†**^	0.9–1	0.72^**†**^	0.71–0.98^**†**^
Pathways	VEGF Interferon Interleuki Viral genome Toll-receptor NF-κB T-cell; B-cell Platelet	Interferon Interleukin	Interleukin NF-κB T-cell Lung	Interleukin VEGF Epigenetic	NA	NA	T-cell Interleukin	Interleukin GrowthFactor

*HC* healthy control, *Mod* moderate COVID-19, *Sev* severe COVID-19.

^†^ AUC scores predicting severity (ARDS +).

*AUC scores from cross-study predictions.

**Figure 1:**
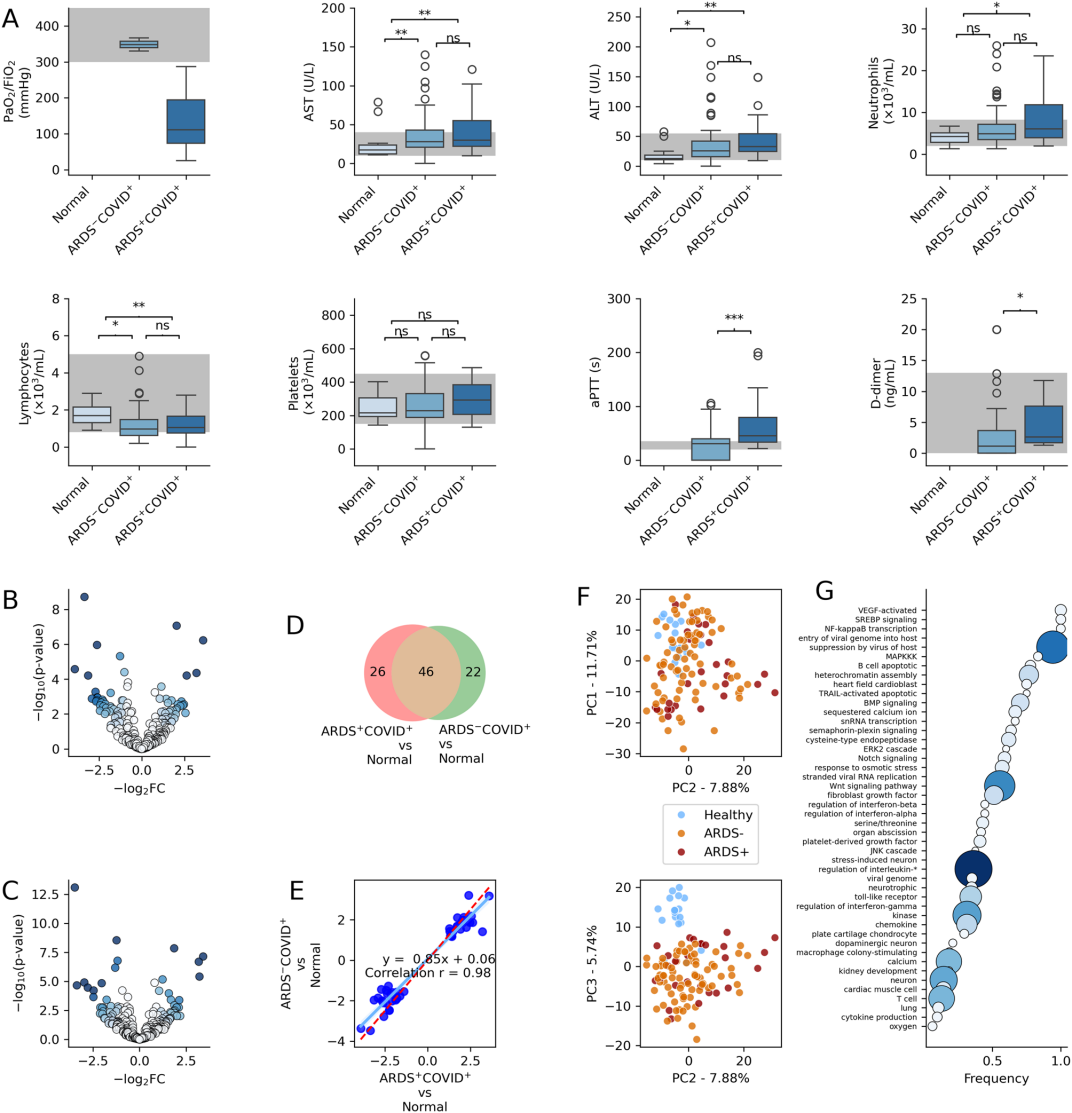
Expression of miRNA and clinical biomarkers in COVID-19 and non-COVID-19 general population donors. (**A**) Traditional clinical biomarkers were affected by COVID-19, including the PaO2:FiO2 ratio, AST and ATL levels, neutrophil and lymphocyte counts, and aPTT duration. The normal reference range is indicated in gray. (**B**) Volcano plot for ARDS^+^COVID^+^ patients vs normal or non-COVID-19 plasma donors. The depth of color is proportional to the product of log_2_ fold change values and –log_10_ p-values. (**C**) Volcano plot for moderate ARDS^-^COVID^+^ vs normal or non-COVID-19 plasma donors. (**D**) Venn diagram showing overlap between the two sets of comparisons (ARDS^+^COVID^+^ vs Normal and ARDS^-^COVID^+^ vs normal). (**E**) Correlation among shared differentially expressed markers for ARDS^-^COVID^+^ vs normal and moderate ARDS^-^COVID^+^ vs normal sets (**F**) Principal component analysis of microRNA dynamics. Normal, ARDS^+^COVID^+^ and ARDS^-^COVID^+^ are represented with blue, red and orange dots respectively. (**G**) Top enriched gene ontology (GO) categories for ARDS^+^COVID^+^ vs Normal set. Frequency was calculated by dividing hit times with total hits within category in GO-basis database. The color and size of the marker represents hit counts. Dunn test p-values < 0.05 and ≥ 0.005 are indicated by *, < 0.005 and ≥ 0.0005 are indicated by **, and < 0.0005 are indicated by ***. The letter ‘ns’ denotes non-significant differences.

For our study, all samples were collected at the time of admission into the hospital when the patient received a COVID positive test and approximately 2–3 weeks before their severity was categorized. The PF ratio measurements were only available for patients with severe COVID and were intubated (Supplementary Table S1). The median PF ratio for severe COVID patients was 111 mmHg, with an interquartile range of 73–194 mmHg. The occurrence of out-of-range values for aspartate transaminase (AST), alanine transaminase (ALT), neutrophils, lymphocytes, platelets, and activated partial thromboplastin time (aPTT) was higher in the severe COVID group compared to the moderate COVID group and the pre-COVID normal subjects. Specifically, the percentages of out-of-range values for AST, ALT, neutrophils, lymphocytes, platelets, and aPTT were 41, 22, 32, 26, 11, and 74% in the severe COVID group, 29, 15, 18, 33, 7, and 35% in the moderate COVID group, and 11, 6, 0, 0, and 6% in the pre-COVID normal subjects, respectively (Table S1 and Fig. 1).

Similarly, COVID-19 patients, especially severe (ARDS^+^) cases, showed statistically significant increases in AST (p-value < 0.005), ALT (p-value < 0.005), and neutrophil (p-value < 0.05) levels, as well as significant decreases in lymphocyte counts (p-value < 0.005) compared to healthy volunteers (Fig. 1A). Similar observations were also reported in Togami et al.^12^ study. Most CBC and clinical chemistry biomarkers, including AST, ALT, neutrophils, and lymphocytes, were unable to differentiate between severe and moderate COVID groups, with the exceptions of aPTT and d-dimer, which showed higher values in severe COVID (Fig. 1A).

### miRNA dynamics

In our study around 18% to 35% of the reads in our Illumina libraries consist of miRNAs. To ensure comparability with other published works for meta-analysis, our analysis focused on miRNA and did not encompass other cfDNA, RNA or proteins present in the collected plasma samples. Severe SARS-CoV-2 infection led to differential expression of 72 miRNAs when compared to healthy volunteers (p-value < 0.05 and > 2.25-fold change in expression; Fig. 1B). Among moderate COVID-19 patients and healthy controls, 68 miRNAs were differentially expressed (Fig. 1C), with 46 of these miRNAs overlapping with the severe COVID vs normal case (Fig. 1D). The shared differentially expressed miRNAs exhibited highly correlated expression (Pearson correlation r = 0.98; Fig. 1E), suggesting that severe and moderate COVID-19 share a similar miRNA transcriptome response. Principal component analysis (PCA) further illustrates the close relationship between severe (ARDS^+^) and moderate (ARDS^-^) COVID (Fig. 1F). Conversely, the PCA analysis demonstrated a clear separation of normal and COVID samples, with the greatest separation observed along the PC3 axis. This axis accounted for 5.74% of the total variance among all miRNAs (Fig. 1F).

The top differentially expressed miRNAs identified in the severe COVID vs normal comparison included hsa-miR-150-5p, hsa-miR-423-3p, and hsa-miR-381-3p, among others (Supplementary Table S3). The robustness of these results was confirmed using 100 bootstrapping iterations of patient samples (Supplementary Fig. S1), and many of the same miRNAs were also found to be differentially expressed in the moderate COVID (ARDS^-^COVID^+^) vs normal comparison (Fig. 1 and Supplementary Table S3). In addition, the study observed a strong correlation between the early and later wave of COVID-19 disease responses (r = 0.78 for all markers regardless of DE or not, Supplementary Fig. S2). We've observed only a 10–30% overlap (Supplementary Tables S3–S5) of DE genes defined across different studies.

**Figure 2:**
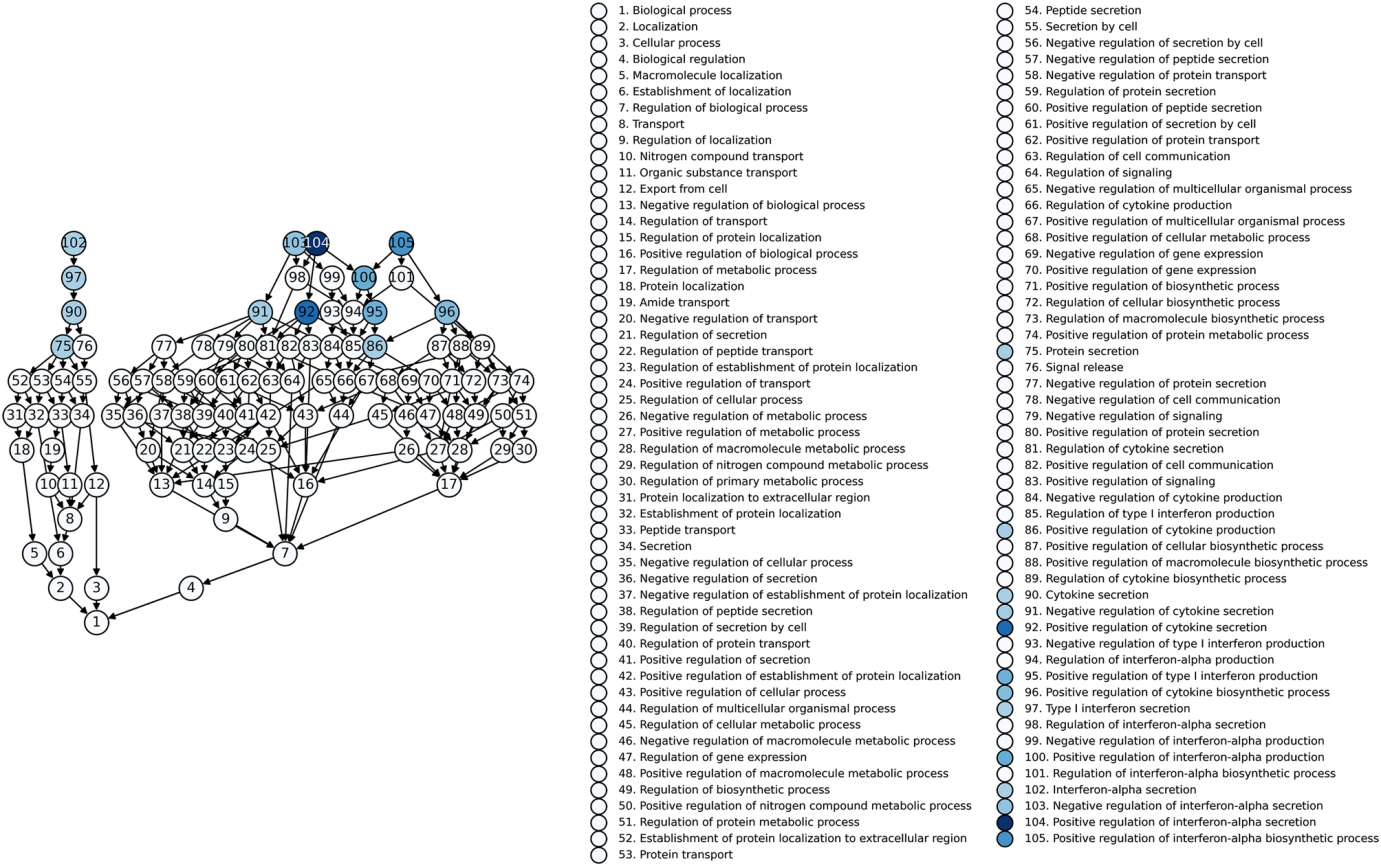
Interferon alpha pathways involved in severe COVID pathogenesis. Colors indicate p-values from Fisher’s exact test. Additional interferon beta and gamma pathways and more details such as p-values can be found in supplementary figures and tables.

The predicted functional roles of these identified miRNAs (ARDS^+^COVID^+^ vs normal set) were assessed using GOEA, which identified 1492 enriched GO pathways (FDR corrected Fisher’s exact p-value < 0.05, Supplementary Table S6). These pathways were then clustered based on keywords (e.g. apoptosis) or sub-terms (e.g. DNA damage response), and the frequency of enrichment for each keyword/sub-term was calculated (Fig. 1G). The top GO pathways included vascular endothelial growth factor (VEGF) signaling, which promotes angiogenesis and vascular permeability; SREBP signaling, which is involved in fatty acid metabolism; NF-κB transcription, which plays a role in inflammation, immunity, and cell survival; interleukin-mediated signaling that play important roles in the immune system, suppression by virus of host; regulation of interferon-beta, alpha, and gamma that are known to be involved in viral and COVID responses, among others; the JNK pathway regulates gene expression and cellular functions involved in inflammation, immune response, and cell survival. The interferon alpha pathway is shown in Fig. 2. Supplementary Figs. S3–S7 provide further network elaboration of these pathways, including the interrelationships between different categories and subcategories. Different studies tend to reveal overlapping pathways, the interferon and multiple cytokines were repeatedly detected (Table 1).

In our study, GOEA revealed pathways specifically related to the viral genome and its activities. One of the top (4th highest in fold change values, 5.9 fold, logFC = 2.56) differentially expressed miRNAs, hsa-miR-1246, was predicted to be directly involved in targeting the SARS-CoV-2 viral genome^20^. Two other miRNAs, hsa-miR-141-3p, hsa-miR-628-3p and hsa-miR-193a cluster were also predicted to have similar functions^4,21^. Supplementary tables and figures (Supplementary Table S6, Fig. S7) provide further information on specific pathways related to the viral genome or viral activities. We also utilized another tool, miEAA^22^, for over-representation and enrichment analysis. However, we did not uncover any significant findings, except for the confirmation that many of the top differentially expressed genes in COVID disease are specific to blood tissues (over-representation analysis ORA p-value = 2.8e-9).

### Building and validating machine learning models for distinguishing COVID disease and severity

**Figure 3:**
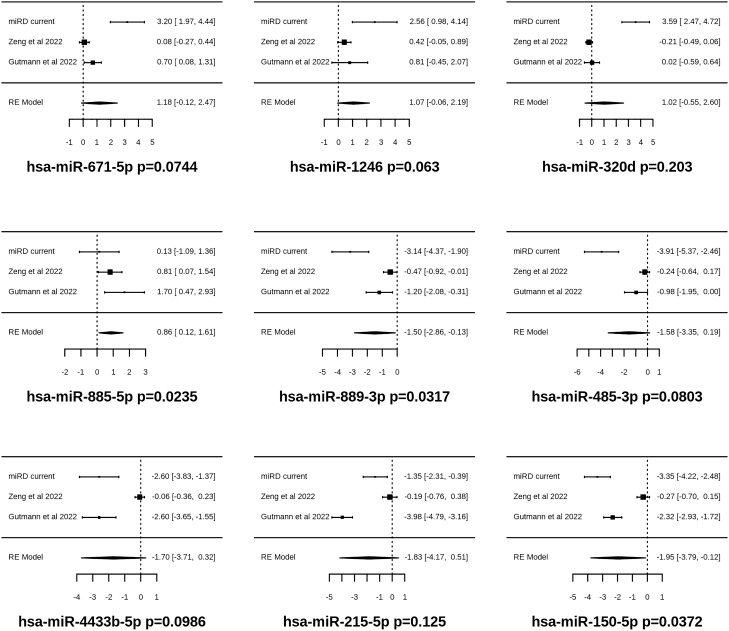
Forest plots for meta-analysis of three independent studies on miRNA dynamics upon SARS-COV2 infection. Results are sorted by effect sizes, and the most up regulated (4) and down regulated (5) miRNAs were shown. More details of meta-analysis can be found in supplementary information.

We investigated the feasibility of utilizing top differentially expressed (DE) miRNAs as features for constructing machine learning models to predict COVID disease or severity, achieving prediction accuracies greater than 0.92 and AUC scores greater than 0.95 (Supplementary Fig. S8). However, while this approach has been widely used in published studies, there are potential limitations to its generalizability in other studies because of sample size limitations, batch effects and variations in sample and sequencing library preparation (Supplementary Fig. S9). Therefore, we propose that building machine learning models based on meta-analysis of multiple studies would enhance the robustness and reliability of these models, especially given the increasing availability of data and recent publications in this field. In addition, computational tools such as ExhauFS were employed to identify top ranked features for machine learning predictions^16^. For model development and validation, our study (n = 18, 27 for disease, and n = 77, 27 for severity) served as the training set, while the study by Zeng et al. (n = 61, 48 for disease and n = 52, 48 for severity) was utilized as the filtration set, with the Guttman Study (n = 12, 18 for disease and n = 18, 18 for severity) and Garcia Study served as validation sets (n = 13, 15 for severity, with no healthy controls). Figure 3 illustrates the top 4 up-regulated and top 5 down-regulated miRNAs ranked based on marker effect (logFC) sizes.

**Figure 4:**
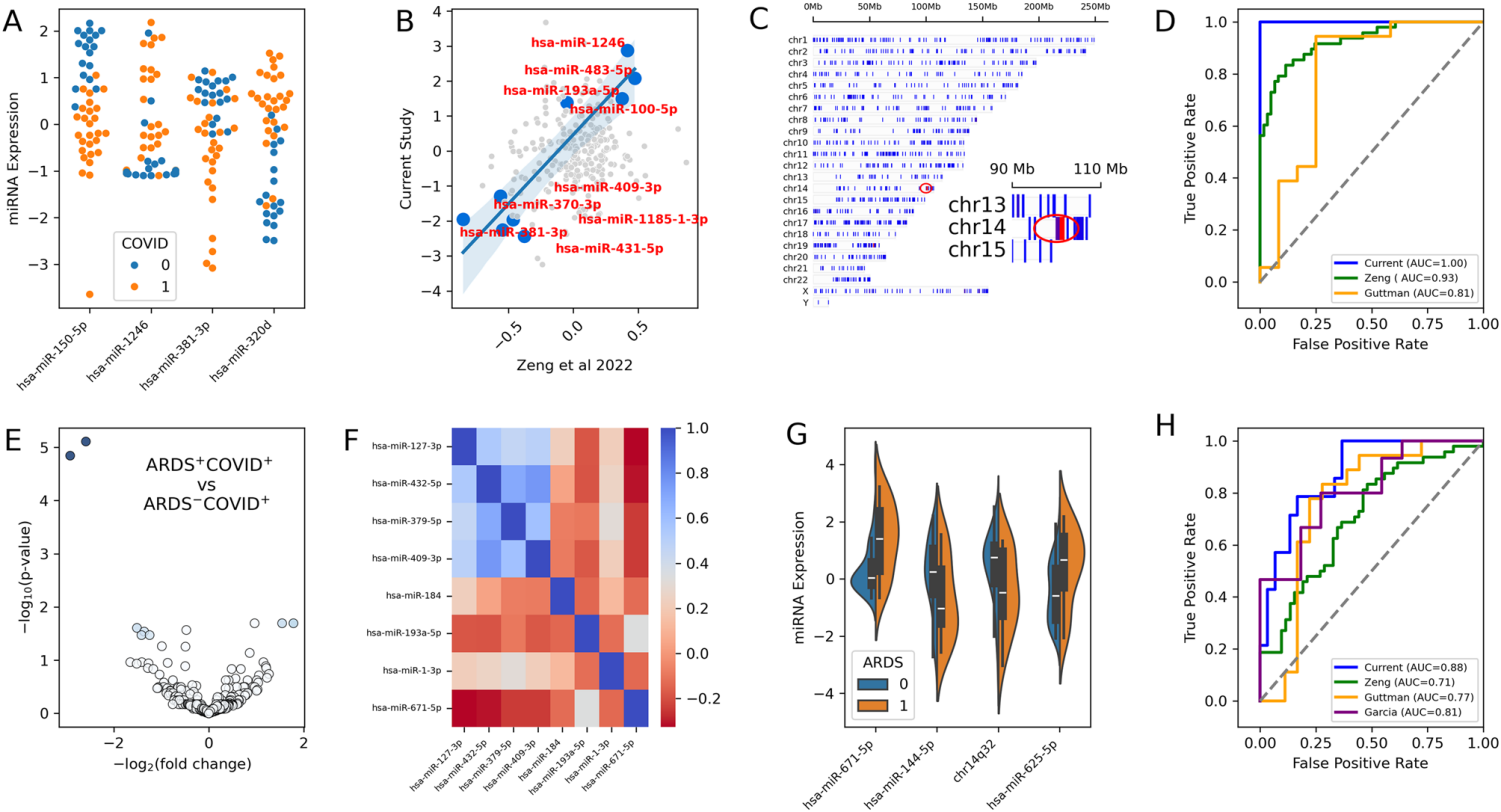
Model feature selections and building for COVID disease (**A**–**D**) and severity (**E**–**H**). (**A**) Swarmplots for model markers distinguishing COVID and healthy. Orange represents COVID, blue represents healthy controls. (**B**) Correlation of current study with published studies Zeng et al. 2022. All significantly downregulated and correlated markers are in the chr14q32 cluster, indicated as red in plot (**C**). (**C**) miRNA density plot along human chromosomes, color was coded based on number per Mb, with red indicating higher density and blue indicating lower density. (**D**) Model performance AUC curves for current (blue), green (Zeng et al.^9^) and Guttman (Yellow) studies. (**E**) Volcano plot for severe ARDS COVID vs moderate COVID. The depth of color is proportional to the product of log2 fold change values and –log10 p-values. (**F**) Correlation plot of top DE markers differentiating severe and moderate COVID. (**G**) Violinplot for model markers distinguishing severe COVID and moderate. Orange represents severe COVID, blue represents moderate COVID. (**H**) AUC curves for COVID severity prediction in current (blue), Zeng et al.^9^ (green), Guttman (yellow), and Garcia (purple) studies.

We selected the top three miRNAs (hsa-miR-150-5p, hsa-miR-1246, and hsa-miR-381-3p) from meta-analysis for building machine learning models to predict COVID disease (Fig. 4A). In this approach, we combined data from three separate studies and used inverse variance weighting to identify the most strongly regulated miRNAs and top correlated markers (Supplementary Fig. S10, Tables S4–S8). We further performed a linear regression and correlation analysis using the current study and the Zeng et al.^9^ study (the one with the highest number of samples), which revealed a group of consistently down-regulated miRNAs including hsa-miR-381-3p, hsa-miR-431-5p, hsa-miR-370-3p, among others (Fig. 4B). On the other hand, the top up-regulated miRNAs included hsa-miR-1246, hsa-miR-483-5p among others (Fig. 4B). Interestingly, all the correlated down-regulated miRNAs among the two studies were from an evolutionarily conserved cluster located at chromosome 14 at the physical bin of 14q32 (Fig. 4C). This miRNA cluster has previously been identified to be involved in various cancer disease responses^23,24^, and, to the best of our knowledge, our study is the first to robustly associate this cluster with COVID response. The resulting model achieved high AUC scores of 1.0 for within study classifications, 0.93 for the classification of the Zeng et al. study, and 0.89 for the Guttman et al. study which did not include age and sex information for individual patients (Fig. 4D). The sensitivity/true positive rate (TPR) are 0.96 for current, 0.56 for Zeng and 0.89 for Guttman. The specificity/true negative rate (TNR) are 1.0 for current, 1.0 for Zeng and 0.75 for Guttman (Supplementary Table S9).

While classifying patients into COVID or non-COVID based on miRNA data might have limited clinical application, we further investigated the use of miRNA features to predict disease severity or prognosis (ARDS^+^ or ARDS^-^). The feasibility of this prediction is based on the observation that our sample collection dates are often 2–3 weeks before the diagnosis of ARDS or ventilation setting dates (Supplementary Table S1, Fig. S11). As previously mentioned, severe COVID (ARDS^+^) and moderate COVID (ARDS^-^) patients exhibit similarities in miRNA responses (Fig. 1D–F). Consequently, we identified 8 miRNAs that were differentially expressed between the two groups (Fig. 4E,F). Remarkably, 4 of these markers were once again located in the 14q32 cluster, and their expression values showed high correlation (Fig. 4F). We thus explored the possibility of combining 14q32 markers into a single feature by averaging the expression values of 4^+^ miRNAs (Fig. 4G). By selecting the strongest and most consistent DE markers (Fig. S10), and the engineered or combined feature of chr14q32, we achieved good classification and prognosis of whether a patient might develop severe ARDS following COVID infection (Fig. 4H). The model had an AUC score of 0.88 for the current study, 0.81 for the Guttman study, and 0.77 for the Garcia study despite using different sample types (Garcia, serum vs plasma) or lacking sex and age information (Guttman). The AUC score for the Zeng et al. study was lower (0.71) with the current modelling, which is likely due to the differences in ethnic compositions, disease severity classification criteria, or library preps (extra PCR step was used in Zeng et al. study). Overall, our models trained on the current study achieved decent predictive power for multiple independently published studies with different sample collection and library preparation procedures, representing a significant advancement compared to previously published but not independently validated models. The sensitivity (True Positive Rate, TPR) and specificity (True Negative Rate, TNR) values are listed in Supplementary Tables S10, S11. The sensitivity values are 0.57, 1, 0.6 and 0.98 for current, Garcia, Guttman and Zeng studies respectively. The specificity values are 0.93, 0, 0.78 and 0.02 for the studies. Different models have variations in these measures with random forest providing slightly better accuracy measures, but at the expense of AUC scores. The lower specificity measures observed in the Zeng study can be attributed to the additional PCR steps in library preparation when compared to our approach. Similarly, the reduced accuracy measures in the Garcia study are likely associated with differences in sample types (plasma vs. serum).

### Comparisons of our key findings to independently published studies

To put our study into the context of related works, we summarized the population sizes, ethnic composition or country of origins, publication meta data (years, journals) and main findings of several recent publications related with COVID miRNA biomarkers into Table 1. We found that a list of key results from our study were independently confirmed with multiple published studies (Supplementary Tables S3–S5). For instance, hsa-miR-150-5p and hsa-miR-423-3p were repeatedly identified to play critical roles in COVID responses in multiple studies. The hsa-miR-150-5p is a known inflammation marker, and was independently identified in Togami et al., Guttman et al., Fernandez et al. and the current study for playing critical roles in COVID disease immune responses. miR-144-3p and miR-144-5p were identified in Made et al. study that distinguishes severe and non-severe COVID. This miRNA was also found to be one of the most significant DE miRNA distinguishing severe (ARDS^+^) and moderate COVID through meta-analysis. Several additional studies (Zeng et al., Fernandez et al., Farr et al.) identified chr14q32 markers (including hsa-miR-423, hsa-miR-370, hsa-miR-369-3p) as the most interesting or discriminating marker in COVID DE analysis or predictive models. To our knowledge, our study is the first to link these findings together and to clearly identify a genomic locus as the site harboring these miRNAs of interest (Fig. 4 and Supplementary Fig. S12).

We used a database of known miRNA-mRNA interactions^25^ to identify the proteins or genes that could be dysregulated due to the DE of miRNAs. Our results showed that 85% of the fibrosis marker genes, 81% of the angiogenesis markers, and 90% of the coagulation markers listed in a recent cell paper^26^ were predicted to interact with our top DE miRNAs (Supplementary Table S12). Moreover, we observed that over 70% of the DE genes (1089/1546) identified in the Togami et al. study was also predicted to interact with our most significantly DE miRNAs. Furthermore, one of the major genetic loci identified through genome wide association analysis (GWAS) (CCR genes)^27^ in a broad study focused on COVID severity were predicted to interact with our hsa-miR-150, hsa-miR-144, hsa-miR-369, among others. Collectively, these results provide interesting links from miRNAs to genes or proteins and known markers for fibrosis, coagulation, and angiogenesis among others.

## Discussion

The pathophysiology of COVID-19 is complex and can result in severe outcomes, such as ARDS and mortality. Severe cases of COVID-19 are associated with higher rates of death in our study (Fisher’s exact test p = 0.0057). While the availability of effective vaccines has reduced COVID-19 mortality rates in many parts of the world, a significant percentage (3–6%)^28^ of people still develop COVID-induced ARDS. The situation is worse in countries where effective vaccines or quality medical care is still not widely available. To effectively plan treatment, biomarkers that can predict disease severity are needed. Predictive biomarkers are also essential as companion diagnostics for new or existing therapies. Circulating miRNAs are ideal biomarkers for these applications as they are transcriptome-regulating biomolecules that are excreted by tissues throughout the body, stably packaged in vesicles or protein complexes, and accessible via routine blood draw^29^.

We observed that SARS-CoV-2 infection significantly changed the circulating miRNA profile in both moderate (ARDS^-^) and severe (ARDS^+^) COVID patients. Of the 4 miRNAs used in our model distinguishing COVID and healthy controls, hsa-miR-150-5p is a master regulator of inflammatory processes and was detected repeatedly by us and other researchers in radiation and various pathological processes^30–34^; hsa-miR-1246 is among the highest expressed miRNAs in the lung, and it was found to be downregulated in response to COVID^4,35^; has-miR-320d is an anti-inflammatory miRNA and was previously associated with COVID responses^36,37^. hsa-miR-381-3p is among the most strongly down-regulated miRNAs in Zeng et al. study, and correlated well with our study (Fig. 2), and it is also physically located inside the 14q32 cluster on human chromosome 14 (Supplementary Fig. S12). We found that all 14 of the most highly correlated miRNAs are both down-regulated and from this 14q32 cluster. MiRNA clustering is a well-known phenomenon^3^ that has been shown to play significant roles in miRNA biogenesis and transcriptome regulation. Future studies that combine all cluster features have the potential to increase the robustness of models even further.

The DE and correlation analyses (Fig. 1) suggest that severe (ARDS^+^) COVID and moderate COVID exhibit very similar (correlation > 0.98 for shared DE markers detected in both sets) transcriptomic responses. Consequently, only a limited number of markers were identified that could differentiate between severe and moderate COVID. Among the eight DE miRNAs, half (4) are from the 14q32 cluster on chromosome 14. The most down-regulated of these is hsa-miR-127-3p, which has been identified as a potential regulator of COVID through BCL6 and cytokine^38^. However, despite being the most strongly down-regulated miRNA even after meta-analysis of three independent studies (Fig. S10), its expression levels vary largely among studies, and the confidence interval for marker effects suggests that this miRNA may not be the best marker to use in machine learning models to achieve generalization across studies. Indeed, it resulted in high training performance but not great generalization (data not shown). We argue that similar issues might have existed in other published studies that used only one set of experiment results for model training and highlights the value of meta-analysis.

We narrowed down the features in our model to four features for predicting COVID severity: (1) hsa-miR-625-5p, is known to predictively target AKT2 to suppress inflammatory responses in human bronchial epithelial cells^39^. (2) hsa-miR-671-5p is potentially involved in increasing apoptosis by downregulating BCL2 protein expression and modulating responses targeting MCL1 and NF-κB1A hub proteins^40^. (3) hsa-miR-144-5p was found to be involved in cytokine and growth factor pathways and was previously used to distinguish between severe and moderate COVID^19^. (4) We included an engineered (mean of multiple miRNAs) feature from the 14q32 cluster on chromosome 14 in our model. This cluster has been previously associated with various types of cancer such as melanoma, ovarian cancer, head, and neck cancer, and more. However, our study is the first to explicitly link this cluster of miRNAs with COVID disease and progression. The 14q32 cluster is an evolutionarily conserved and parentally imprinted region that may play significant roles in aging (Supplementary Table S13, p = 2.15E-5) and disease progressions, including COVID and other conditions.

The GOEA analysis indicates that the identified miRNAs may be associated with pathways involved in COVID-19-induced ARDS leading to fatal respiratory failure. Previous studies have shown that miRNA biomarkers can provide detailed molecular understanding of ARDS and related diseases^41^. Our GOEA analysis also identified several pathways that could be implicated in SARS-CoV-2 pathogenesis. The c-Jun NH2-terminal kinase (JNK) signaling cascade, which can lead to inflammatory responses, cell proliferation, survival, or death, has been shown to play a critical role in SARS-CoV infection^42^ and has been implicated in SARS-CoV-2-induced apoptosis^43^. Our analysis identified several enriched GO terms associated with the JNK cascade (JUN, JNK, MAPK; Supplementary Table S6, Fig. 1G). SARS-CoV-2 infection may lead to upregulation of the p38 MAPK pathway due to loss of ACE2 activity upon viral entry and by direct viral activation^44^. We identified nine GO terms associated with the pathway, which could result in upregulation of inflammatory cytokines, such as IL-6 and TNF-α, and contribute to severe cardiac and pulmonary injury in COVID-19 patients.

Consistent with previous research, cytokines have been observed to play a role in the severity of SARS-CoV-2 and related coronaviruses^45,46^. Our findings of increased cytokine levels in severe COVID-19 patients are consistent with this, and GOEA identified 84 GO terms (Supplementary Table S5, Fig. 1G) associated with interleukin signaling pathways and secretion. Studies have reported abnormal levels of various interleukins, including IL-1, IL-2, IL-4, IL-10, IL-12, IL-13, and IL-17, which is also consistent with our GO analysis. Toll-like receptors (TLRs) may contribute to the failure of viral clearance and subsequent development of severe secondary consequences. TLR activation causes the production of innate pro-inflammatory cytokines (IL-1, IL-6, TNF-α) and type I IFN-α/β, which are essential for anti-viral responses. GOEA predicted that these pathways may be perturbed by the identified miRNA. GOEA also suggested that type I interferon signaling pathway and interferon-γ-mediated signaling pathway may be differentially regulated in COVID-19. Recent studies suggest that deficiencies in interferon signaling are correlated with worse outcomes in COVID-19 patients^12,47^.

Predicting outcomes in different studies can be challenging due to several factors such as sample types, collection devices, and library preparation steps, among others. These factors can significantly affect the abundance and quantity of biomarkers, leading to high variability and overlapping roles among different miRNA biomarkers. In this regard, our study explored the complexity of predicting outcomes based on one study from another and found that the variability within groups but across studies, such as healthy controls, can be greater than the variability among groups but within studies (Supplementary Fig. S9). Certain published studies did not provide sufficient public data, which renders meta-analysis infeasible. Moreover, there are considerable differences in the software and analysis pipelines employed across different studies, as well as disparities in the p-value and logFC cut-offs that are utilized. Consequently, the differentially expressed (DE) genes defined in one study may not align directly with those defined in another study. We've observed only a low to moderate overlap (Supplementary Tables S3–S5) of DE genes defined across different studies. Despite these challenges, we managed to employ meta-analysis and correlation analysis to identify consistent patterns and to construct machine learning models that demonstrate robust performance. We found that Logistic Regression stands out for its superior AUC, F1-score, and Kolmogorov–Smirnov s statistic, suggesting a good trade-off between the various measures of model performance. SVM also presents as a good model with moderate-to-high values across different metrics. Random Forest and XGBoost are prone to overfitting data from our study given their perfect scores but reduced performance in the other studies (Supplementary Tables S9–S11).

Several of our study's findings such as key miRNAs and prediction models were validated through multiple independent research, highlighting the potential of our approach (meta-analysis) to identifying DE miRNAs, pathways, and developing models, and providing insights for future studies in this field. The circulating miRNAs identified in our study have high predictive value and provide a comprehensive picture of patient pathogenesis. This detailed understanding of the disease could help physicians make informed decisions regarding treatment planning and guide the development of new therapeutics while monitoring their effectiveness.

## Materials and methods

### Clinical specimen

Adult patients hospitalized in Erie County, NY, with PCR-confirmed COVID-19 between March and November 2020 were retrospectively identified by Discovery Life Sciences (DLS; Huntsville, AL). Plasma samples were collected within the initial seven days of hospitalization. Demographic and clinical profiles, encompassing laboratory data, were extracted from discharge summaries. The collection adhered to a protocol approved by the Institutional Review Board at Advarra, Inc. (IRB00000971). For comparative analysis, plasma samples and corresponding clinical data were procured from a pre-pandemic general adult volunteer population without known respiratory illnesses (July 2018 to December 2018), sourced from BioIVT (Westbury, NY). The collection of these normal samples adhered to a protocol approved by the Institutional Review Board at WCG™ IRB. (IRB00000533). From this pool, 18 samples were selected to match the COVID-19 patients' age and sex distribution. Informed consent was obtained from all participants in both cohorts, and venipuncture into Vacutainer® tubes containing EDTA facilitated blood collection, followed by plasma separation. Adherence to relevant guidelines, regulations, and the Declaration of Helsinki was maintained throughout the procedures for both cohorts. Rigorous de-identification measures were applied to patient samples from both cohorts to uphold privacy and confidentiality. The study was conducted according to the guidelines for the use of human subjects’ materials of the “Declaration of Helsinki.”

### miRNA extraction and sequencing

Plasma samples were confirmed to have absorbance value less than 1.2 A.U. at 415 nm, corresponding to < 0.3% hemolysis^48^. Circulating miRNA was isolated from 100 µL of plasma using the miRNEasy Serum/Plasma Advanced Kit (Qiagen). Sequencing libraries were prepared using the QIAseq miRNA Library Kit (Qiagen), with 5.8 µL of miRNA extracts as input, a 1:10 dilution of the 3’-adaptor, a 1:5 dilution of the 5’-adaptor, a 1:10 dilution of the RT primer, and 22 amplification cycles. Library concentrations were determined via Bioanalyzer (2100 Electrophoresis Bioanalyzer, Agilent). Libraries with an adaptor dimer peak (~ 160 nt) at least five times greater than the library peak (~ 180 nt) were not sequenced. miRNA counts for 2 nM samples were determined via next-generation sequencing (NextSeq 550, Illumina) using 76 read cycles. Demultiplexing, trimming (read lengths between 18 and 40 bp, 5’-end base quality ≥ 30, read score ≥ 20, and 3’-end adaptor sequence to trim of AACTGTAGGCACCATCAAT), and miRNA alignment (using “Homo sapiens/hg19” as the species) was performed using BaseSpace (Illumina), using the Small RNA v1.0.1, FASTQ Toolkit v2.2.0, and FASTQ Generation v1.0.0. Sequencing samples with less than 400,000 total reads were excluded from analysis.

### Statistical analysis

Raw sequencing counts were normalized by total library size to obtain the reads per million (RPM), then by quantile normalization of the log_2_ RPM. Differential expression analysis was performed in R (version 3.4.3) using the limma and voom software packages (version 3.28.10)^49^. A total of 100 bootstrap samplings were done to test/confirm the reproducibility of top DE miRNA results. Each miRNA with average sequencing counts > 5 RPM were selected and COVID-19 patients were sorted into two groups based on arterial oxygen (PaO2) and fraction of inspired oxygen (FiO2), PaO_2_:FiO_2_ ratio (or PF ratio). Specifically, patients with PF ratio less than 300 mmHg is considered to be the class of acute respiratory distress syndrome (ARDS^+^), patients with PF ratio greater than 300 mmHg or who were not deemed necessary to have measured PF ratio values were considered to be in the non-ARDS group. The clinical data such as AST, ALT, neutrophil and lymphocyte counts, and aPTT duration, and d-dimer values were compared between the three groups. Dunn's test for multiple comparisons was performed, and the p-values were adjusted using the Holm's method.

Differentially expressed miRNAs were also subjected to Gene Ontology Enrichment Analysis (GOEA). GOEA was performed^50,51^ on these selected miRNAs by identifying miRNA-gene interactions using miRTarBase^52,53^ and miRWalk^25,54^. For each GO term in the “biological process” namespace, the genes associated with the GO term were identified using Homo sapiens GO annotations (http://current.geneontology.org/products/pages/downloads.html). Enrichment was calculated using Fisher’s exact test, as previously described^55^. Statistical differences in laboratory and clinical data were calculated with the Mann–Whitney *U* test for two cohort comparisons and the Kruskal–Wallis test followed by Dunn’s multiple comparison test for three cohort comparisons.

We conducted a meta-analysis to investigate the effects of miRNA of interest, measured as the log_2_ transformed fold change (logFC) and standard error, using data from three independent studies (Gao et al. current, Zeng et al. and Gutmann et al.)^9,10^. The logFC marker effects and standard error data from each study were obtained using unified pipeline (limma-voom, as detailed above) and combined for further analysis. We used the ‘rma’ function from the R metafor package to fit a mixed-effects model to the combined data, which implemented the DerSimonian-Laird method for inverse variance weighting. We then computed the estimates and p-values for each marker to identify the effects of the biomarker of interest. Additionally, we re-analyzed the datasets (GSE182152) from Togami et al.^12^ using our pipeline to identify DE mRNAs which allowed us to assess the percentage of differentially expressed genes that are predicted to interact with differentially expressed miRNA. While our study does not constitute a comprehensive review analysis, we have nonetheless adhered to the PRISMA2020 guidelines, providing details on selection criteria, rationale, methods, and results whenever relevant.

Based on the results of the meta-analysis, we selected the top markers that differentiate the conditions of interest (COVID vs Healthy, ARDS vs non-ARDS) and built machine learning models with our data (and only our data) using the logistic regression (classification) algorithm with the Python scikit-learn module. We performed feature engineering by combining correlated chr14q32 cluster miRNAs into one feature. To validate the performance of our model, we utilized data from independently published studies and plotted the ROC_AUC.

Furthermore, in our pursuit of enhancing our results, we harnessed the power of an exhaustive feature selection tool, known as exhauFS, to refine and augment both our feature selection and model choices. In conducting this analysis, we leveraged our own data as the training set, utilized the Zeng et al. study as a filtration set, and employed the remaining studies as validation sets. Throughout our exploration, we delved into various methods including XGBClassifier, Support Vector Machines (SVM), and Random Forest. We computed a range of evaluation metrics such as sensitivity, specificity, precision, F1-score, Kolmogorov–Smirnov statistic, permutation p-values, among others. We ultimately opted for Logistic Regression as the preferred modeling approach for our COVID disease models. Additionally, we explored the application of Bayesian logistic regression, yielding results remarkably consistent with the frequentist version of logistic regression.

### Research involving human participants and/or animals

The research was carried out in accordance with the 1975 Helsinki Declaration. The study protocol was accepted by the Institutional Review Board of Advarra, Inc. (IRB Number IRB00000971) for the COVID study and by Western Copernicus Group (WCG™ IRB00000533) for the Human Normal study. No animals were involved in the study. 

### Supplementary Information

Supplementary Figures.

Supplementary Tables.

Supplementary Legends.

## Data Availability

Raw sequencing files, processed counts and metadata can be obtained from GEO (NCBI) with an accession number GSE240888. Additional data are available in the supplementary materials.
