# Supplementary material for: Circulating miRNA profiles in COVID-19 patients and meta-analysis: implications for disease progression and prognosis
Source: Sci Rep. 2023 Dec 8;13:21656. doi: 10.1038/s41598-023-48227-w (PMC10709343; doi:10.1038/s41598-023-48227-w)
Supplement: Supplementary file 1 — Supplementary Figures. [file 41598_2023_48227_MOESM1_ESM.docx]

Fig S1 Bootstrapping of samples to confirm the robustness of the top DE miRNAs identified through our study


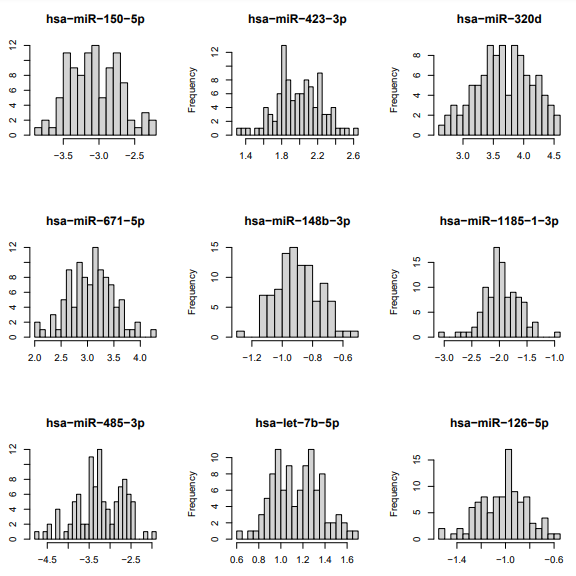


Fig S2 Good correlation of early wave and later wave COVID-19 marker effects (logFC values)


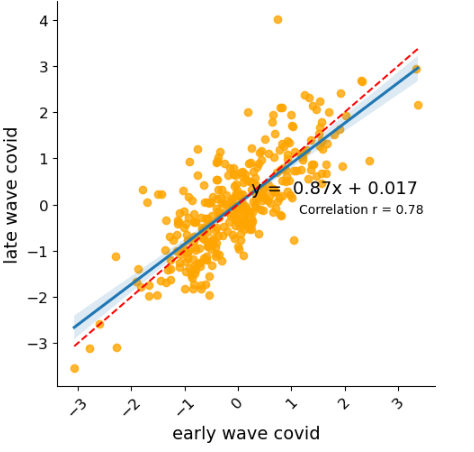


Fig S3 NF-KappaB pathway


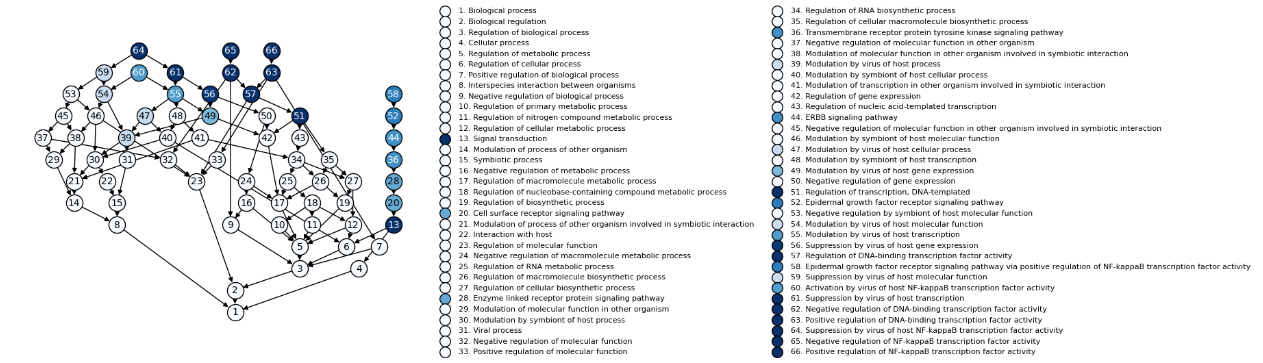


Fig S4 Interferon beta pathway


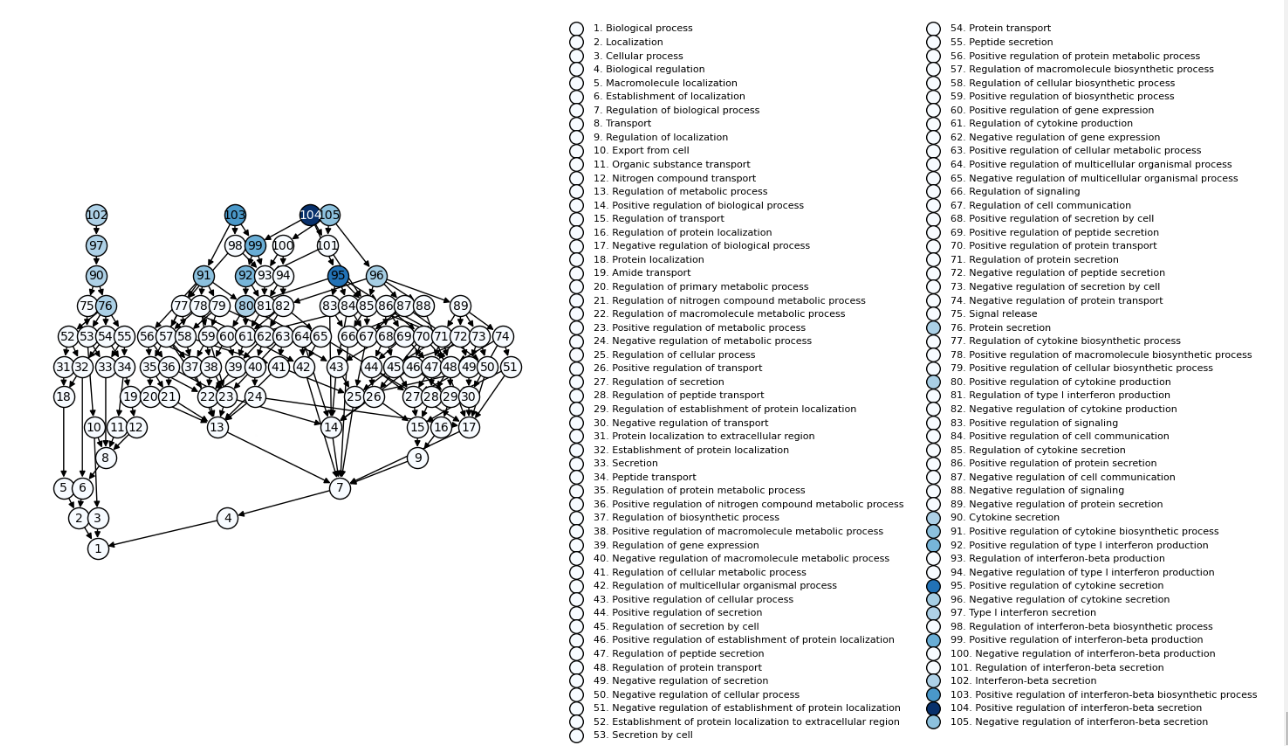


Fig S5 Interferon gamma pathway


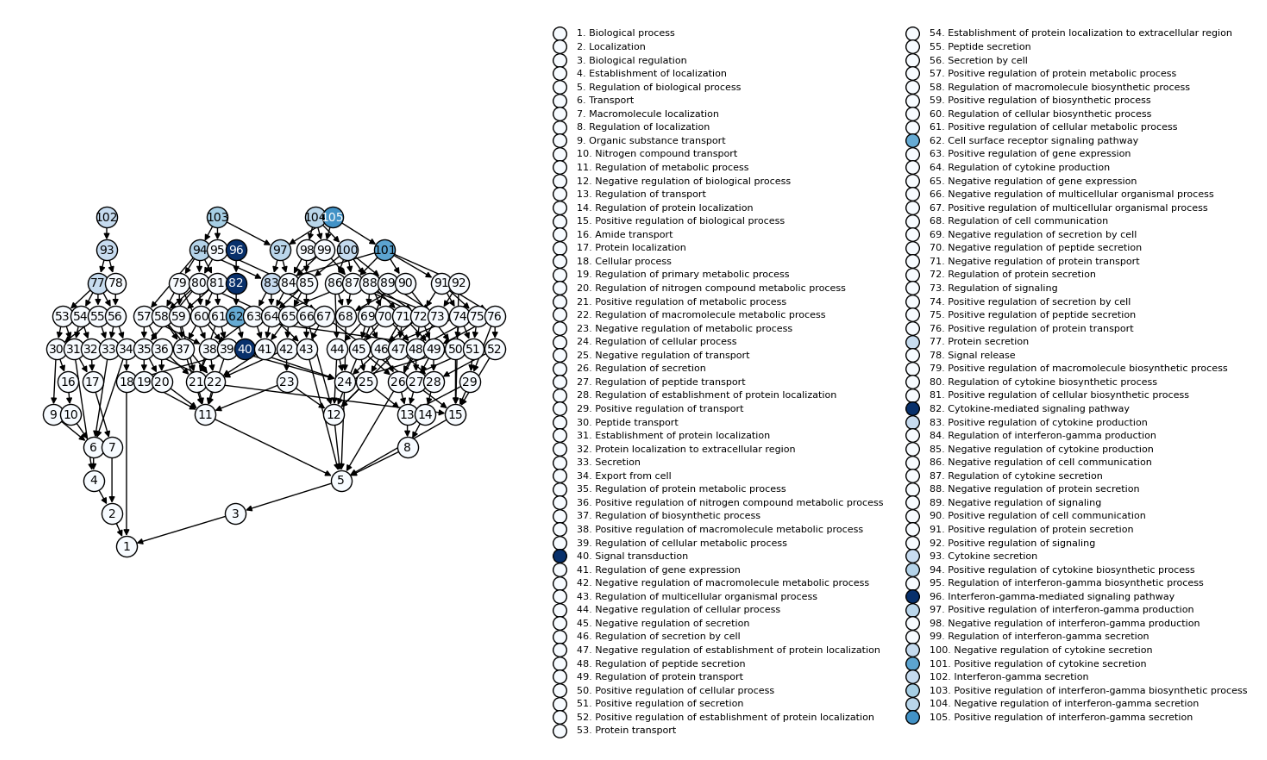


Fig S6 Interleukin pathways


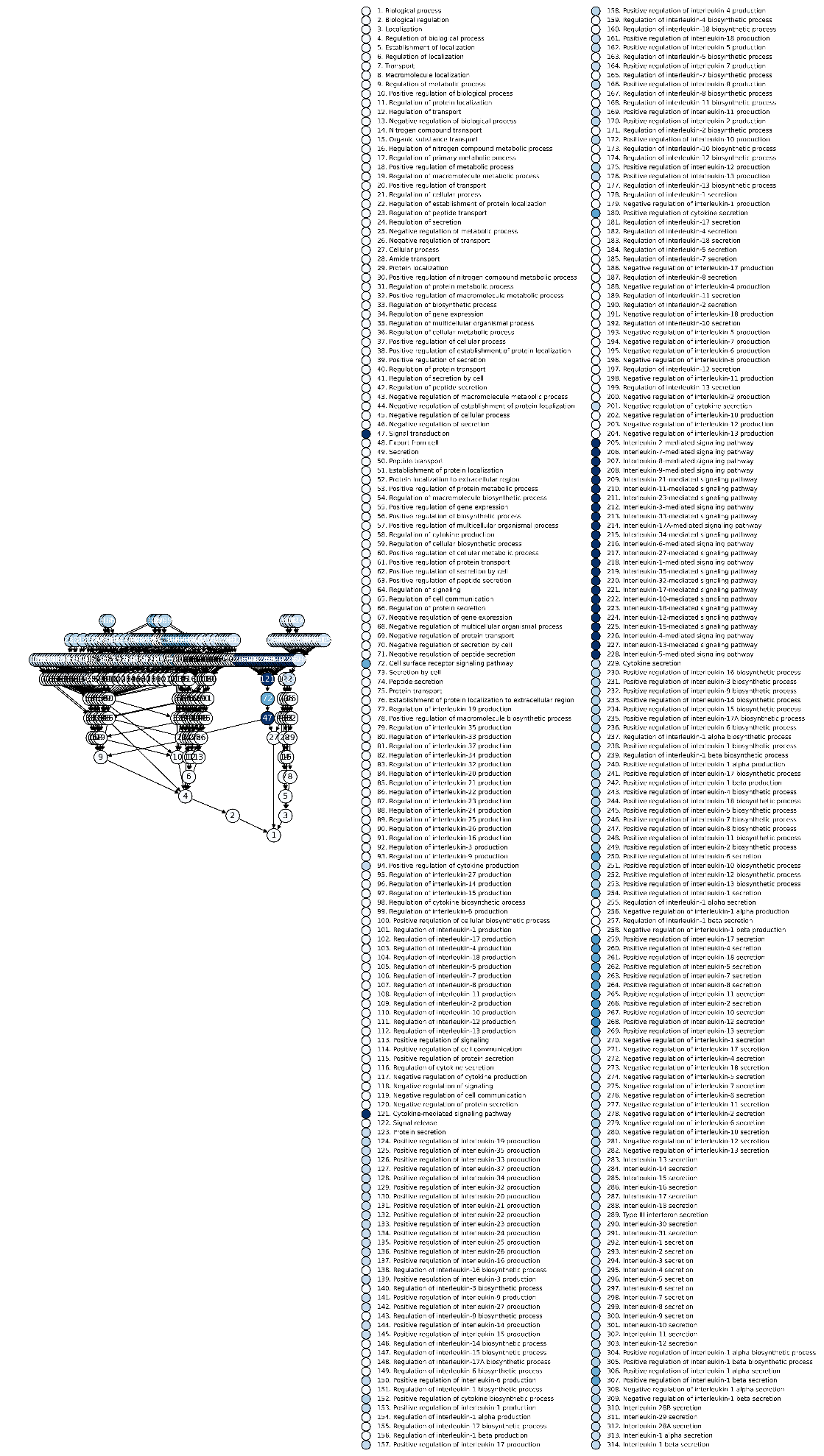


Fig S7 viral genome pathway


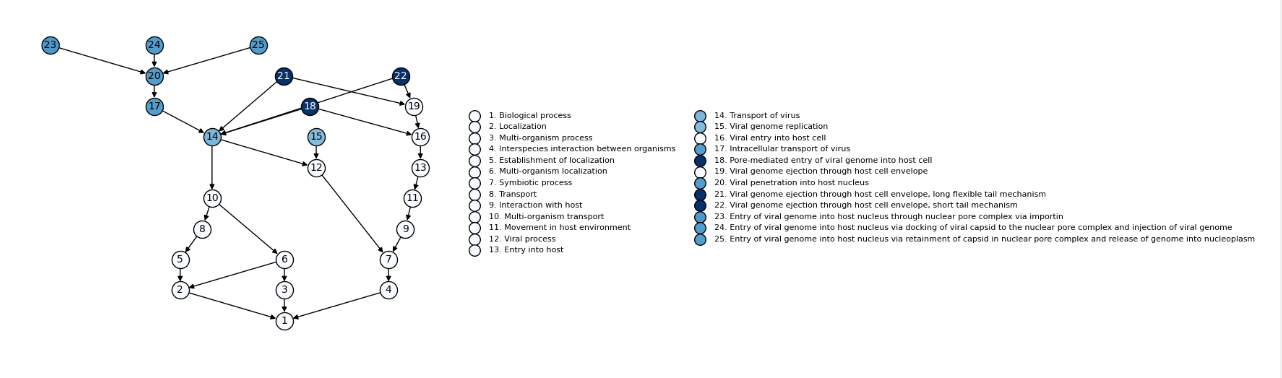


Fig S8 Within study, model performance using top DE miRNAs miR-127, miR-184, and 14q32


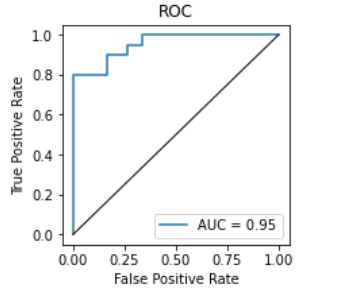


Fig S9 within group vs between group correlation plots of raw counts data


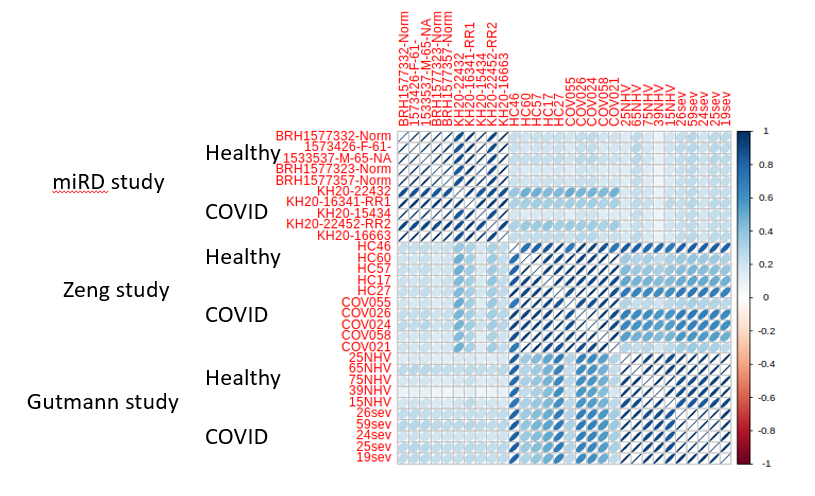


Fig S10 meta-analysis for top DE miRNA for severe vs moderate COVID


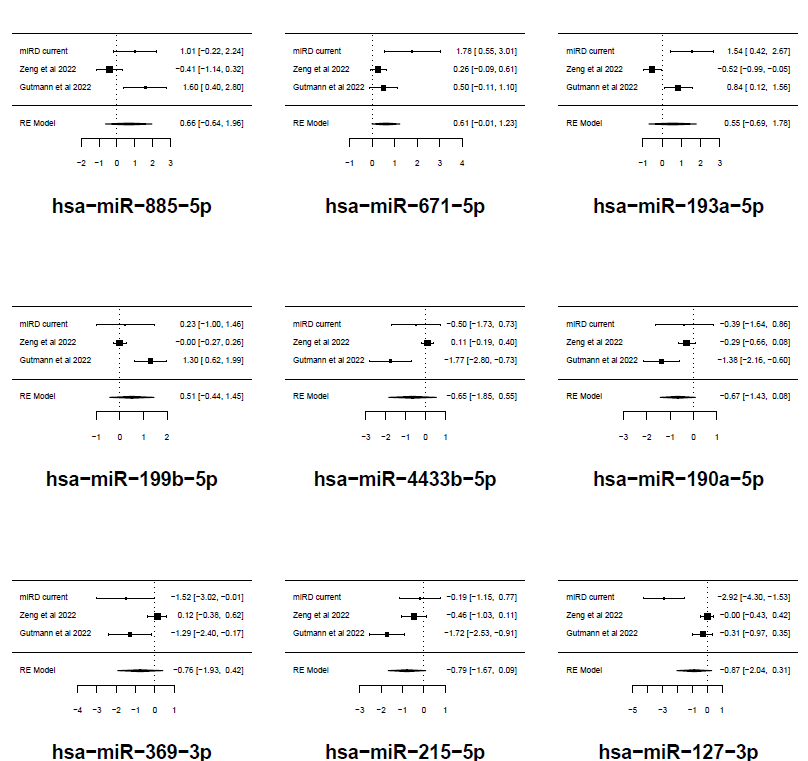


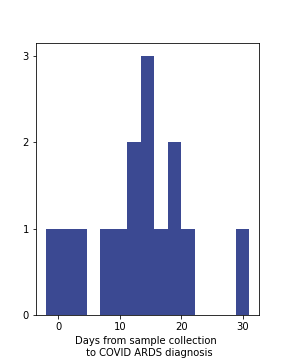
Fig S11 Days from sample collection to ARDS diagnosis or ventilation setting date


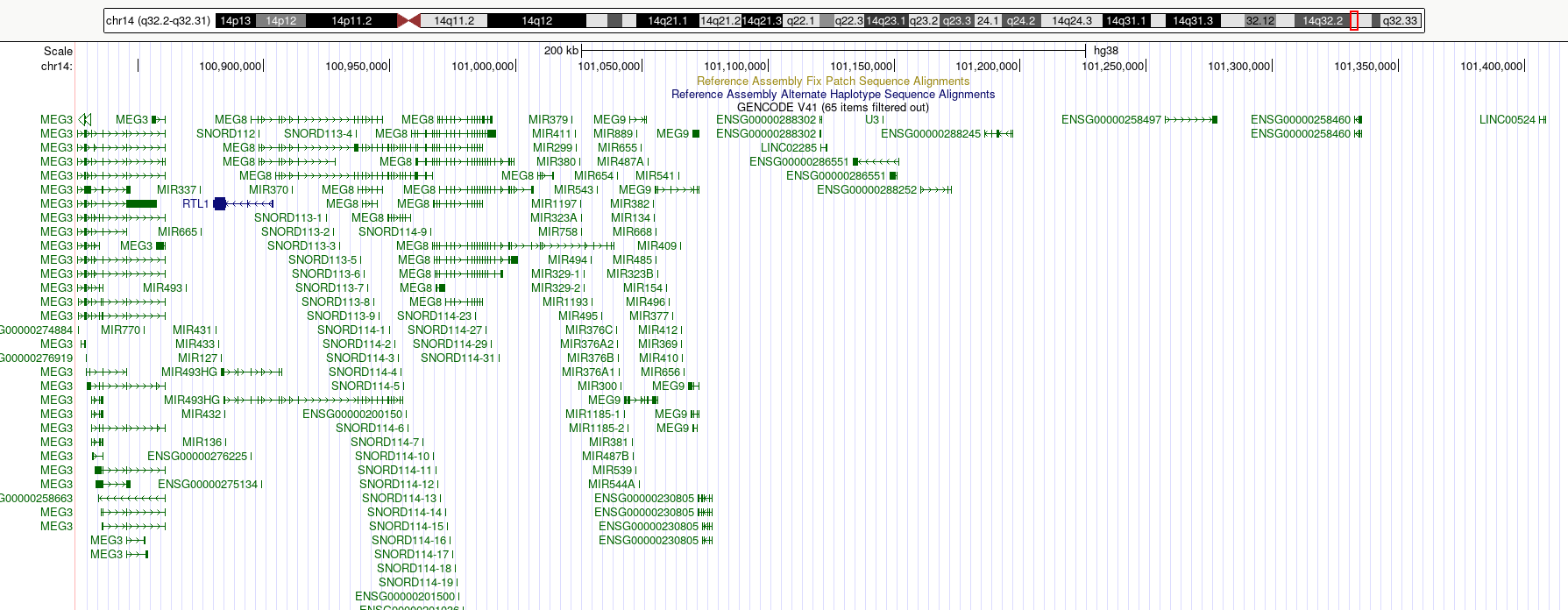
Fig S12 UCSC genome browser zoomed in view of 14q32 cluster of miRNAs
