# Supplementary material for: Circulating miRNA profiles in COVID-19 patients and meta-analysis: implications for disease progression and prognosis
Source: Sci Rep. 2023 Dec 8;13:21656. doi: 10.1038/s41598-023-48227-w (PMC10709343; doi:10.1038/s41598-023-48227-w)
Supplement: Supplementary file 3 — Supplementary Legends. [file 41598_2023_48227_MOESM3_ESM.docx]

**Supporting information**

Table S1 Patient demographics and clinical biomarker data

Table S2 Raw counts for all clinical samples

Table S3 A full list of miRNAs effects (logFC) and p-values for ARDS^+^COVID^+^ vs normal, ARDS^-^COVID^+^ vs normal and ARDS^-^COVID^+^ vs ARDS^+^COVID^+^ comparisons

Table S4 A full list of miRNAs differential expression analysis redone using our software and pipelines for Zeng et al study.

Table S5 A full list of miRNAs differential expression analysis redone using our software and pipelines for Gutmann et al study.

Table S6 Gene ontology enrichment analysis (GOEA) for top DE in ARDS^+^COVID^+^ vs normal comparison

Table S7 Meta-analysis results for COVID vs Healthy comparisons

Table S8 Meta-analysis results for severe COVID vs moderate COVID

Table S9 COVID-19 disease prediction models (Logistic Regression, Random Forest, Support Vector Machine, XGBoost) performance metrics including sensitivity specificity KS statistic AUC

Table S10 COVID-19 disease severity prediction models (Logistic Regression, Random Forest, Support Vector Machine, XGBoost) performance metrics including sensitivity specificity KS statistic AUC

Table S11 COVID-19 disease severity prediction models (Logistic Regression, Random Forest, Support Vector Machine, XGBoost) performance metrics including sensitivity specificity KS statistic AUC without gender and age information

Table S12 Fibrosis, coagulation and angiogenesis marker genes that are predicted to interact with our DE miRNA

Table S13 Gene ontology enrichment analysis using miRnet for chr14q32 cluster miRNA

Figure S1 Bootstrapping of samples (iter=100) to confirm the robustness of top DE

Figure S2 Early wave (alpha 2020) and later wave (delta 2021) marker effects correlations

Figure S3 Network visualization of NF-κB pathway

Figure S4 Network visualization of interferon beta pathway

Figure S5 Network visualization of interferon gamma pathway

Figure S6 Network visualization of interleukin pathway

Figure S7 Viral genome related pathway

Figure S8 Within study model performance using top DE hsa-miRNAs hsa-miR-127, hsa-miR-184, 14q32

Figure S9 Within group and between group correlation plots of raw counts data for three separate studies

Figure S10 Meta-analysis of top DE miRNA for severe vs moderate COVID

Figure S11 Histogram of days from sample collection to ARDS diagnosis or ventilation setting

Figure S12 UCSC genome browser zoomed in view of 14q32 miRNAs
